# Retinal Neurodegeneration in Wilson’s Disease Revealed by Spectral Domain Optical Coherence Tomography

**DOI:** 10.1371/journal.pone.0049825

**Published:** 2012-11-16

**Authors:** Philipp Albrecht, Ann-Kristin Müller, Marius Ringelstein, David Finis, Gerd Geerling, Eva Cohn, Orhan Aktas, Hans-Peter Hartung, Harald Hefter, Axel Methner

**Affiliations:** 1 Department of Neurology, Medical Faculty, Heinrich-Heine University, Düsseldorf, Germany; 2 Department of Ophthalmology, Medical Faculty, Heinrich-Heine University, Düsseldorf, Germany; Institute Biomedical Research August Pi Sunyer (IDIBAPS) - Hospital Clinic of Barcelona, Spain

## Abstract

**Background/Objective:**

In addition to cirrhosis of the liver, Wilson’s disease leads to copper accumulation and widespread degeneration of the nervous system. Delayed visual evoked potentials (VEPs) suggest changes to the visual system and potential structural changes of the retina.

**Methods:**

We used the latest generation of spectral domain optical coherence tomography to assess the retinal morphology of 42 patients with Wilson’s disease and 76 age- and sex-matched controls. We measured peripapillary retinal nerve fiber layer (RNFL) thickness and total macular thickness and manually segmented all retinal layers in foveal scans of 42 patients with Wilson’s disease and 76 age- and sex-matched controls. The results were compared with VEPs and clinical parameters.

**Results:**

The mean thickness of the RNFL, paramacular region, retinal ganglion cell/inner plexiform layer and inner nuclear layer was reduced in Wilson’s disease. VEPs were altered with delayed N75 and P100 latencies, but the N140 latency and amplitude was unchanged. An analysis of the laboratory parameters indicated that the serum concentrations of copper and caeruloplasmin positively correlated with the thickness of the outer plexiform layer and with N75 and P100 VEP latencies.

**Conclusion:**

Neuronal degeneration in Wilson’s disease involves the retina and changes can be quantified by optical coherence tomography. While the VEPs and the thickness of the outer plexiform layer appear to reflect the current copper metabolism, the thicknesses of the RNFL, ganglion cell/inner plexiform layer, inner nuclear layer and the total paramacular thickness may be the best indicators of chronic neuronal degeneration.

## Introduction

Wilson’s disease is an autosomal recessively inherited disorder that leads to copper accumulation and, consequently, to hepatic damage and neuropsychological symptoms [1], [2]. The causative mutations affect the copper-transporting P-type ATPase ATP7B, which regulates the hepatic copper metabolism, leading to impaired biliary excretion and the toxic accumulation of copper primarily in the brain and liver (reviewed in [3] and [4]). Intracellular copper deposits impede inhibitor of apoptosis proteins (IAPs), which eventually causes apoptotic cell death [5]. The clinical presentation varies from predominantly hepatic to predominantly neurologic and shows great heterogeneity regarding severity, age of onset and initial symptoms [6]. Wilson’s disease results in severe disability and death if untreated. The key neurological features comprise extrapyramidal symptoms, ataxia, dystonia, seizures and psychiatric symptoms, such as personality changes, depression and psychosis (reviewed in [7]). Structural changes in the brain of Wilson’s disease patients have been well documented by magnetic resonance imaging (MRI), which has revealed lesions of the basal ganglia, midbrain, pons and cerebellum and widespread cortical atrophy and white matter changes [8], [9]. Histological studies have reported necrosis, gliosis and cystic changes in the brainstem, thalamus, cerebellum and cerebral cortex of Wilson’s disease patients [4]. The functional consequences of these structural changes have been demonstrated in the acoustic, sensory, motor and visual systems and are reflected by disordered multimodality evoked potentials [10]–[13]. Visual evoked potentials (VEPs) have been reported to be abnormal in approximately 50% of symptomatic Wilson’s disease patients [10], [11], [14]–[16]. Common ocular findings of Wilson’s disease include the Kayser–Fleischer ring and sunflower cataracts. Both are due to copper deposition and do not cause visual impairment, suggesting that the observed pathologies in VEPs may be explained by retroocular changes. However, altered flash electroretinograms in Wilson’s disease are indicative of a retinal pathology [12]. Optical coherence tomography is a fast and non-invasive technique and the latest generation of OCT devices is capable of depicting retinal changes at nearly the cellular level [17]–[25]. In this study, we used up-to-date OCT technology to analyze the retinal changes in Wilson’s disease patients. We compared the morphological changes measured by a state-of-the-art spectral domain OCT device with VEPs as functional parameters and correlated these findings with laboratory parameters and a clinical Wilson’s disease score [26].

## Materials and Methods

### Ethics Statement

The work was conducted in accordance with the declaration of Helsinki. Written informed consent was obtained from all patients and the study was approved by the local ethics committee, the “Ethikkommission der Heinrich Heine Universität, Düsseldorf”.

### Patients

We examined 42 patients with Wilson’s disease and 76 control patients without ophthalmologic, inflammatory or degenerative neurological disease. All Wilson’s disease patients were clinically diagnosed following the established criteria [27], underwent longterm follow-up examinations (mean follow up period 10±1 years) and were under therapy with D-penicillamine, trientine, tetrathiomolybdate and/or zinc. The copper and caeruloplasmin concentrations in serum and the 24 h urine copper excretion were measured at the time of the ocular exam and the patients were scored using an established clinical score [26]. All patients underwent formal ophthalmologic exams to rule out confounding ocular pathologies and three eyes were excluded due to central serous retinopathy, vitreomacular traction or paramacular scars.

Patients were consecutively recruited from the outpatient clinic of the department of neurology at the Heinrich-Heine University Düsseldorf, Germany.

### Optical Coherence Tomography

**Figure 1:**
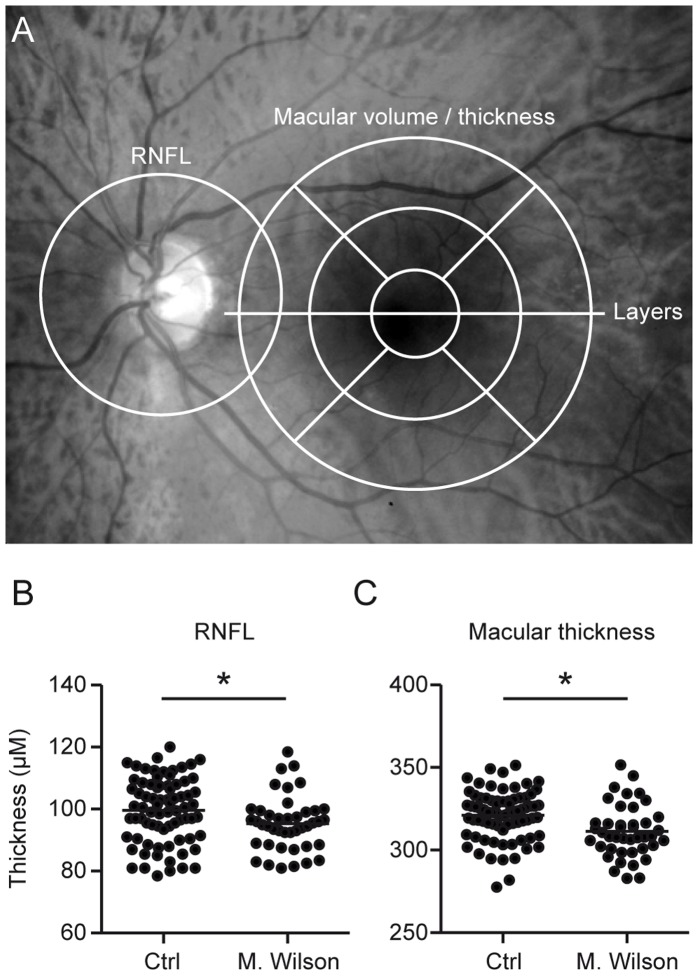
RNFL and mean macular thickness are reduced in Wilson’s disease. A The areas of measurement are marked in an image of the fundus. The RNFL was measured in a circular scan centered on the optic disc and the macular thickness in scans around the fovea. The manual segmentation of the retinal layers was performed in a horizontal scan through the center of the fovea. The scatter plots display the thickness of the RNFL (**B**) and the total macular thickness (**C**). Each point represents the mean of the two eyes of one patient. The mean of all patients is indicated by a horizontal bar. Significant differences are indicated by asterisks (*p<*0.05, two-tailed t-test).

The details of the principles of spectral-domain OCT have been described elsewhere [28]. Using a Spectralis OCT device (Heidelberg Engineering, Heidelberg, Germany) with image alignment eye tracking-software (TruTrack, Heidelberg Engineering, Heidelberg, Germany), we obtained perifoveal volumetric retinal scans consisting of 25 single vertical axial scans (scanning area: 6 × 6 mm, centered at the fovea). To assess the peripapillary RNFL, a circular scan with a diameter of approximately 3.4 mm was performed after manually positioning the center on the middle of the optic disc (figure 1 A). Furthermore, we performed high-resolution horizontal scans through the middle of the fovea. All of the scans were performed using the eye-tracking system. The RNFL measurements and high-resolution single horizontal scans were averaged from 100 images and measurements for volumetric calculations were averaged from 10 scans (Automatic Real Time, ART). All of the scans were of sufficient or good quality (>20 DB) and fulfilled OSCAR-IB quality control criteria for OCT scans [29].

**Figure 2:**
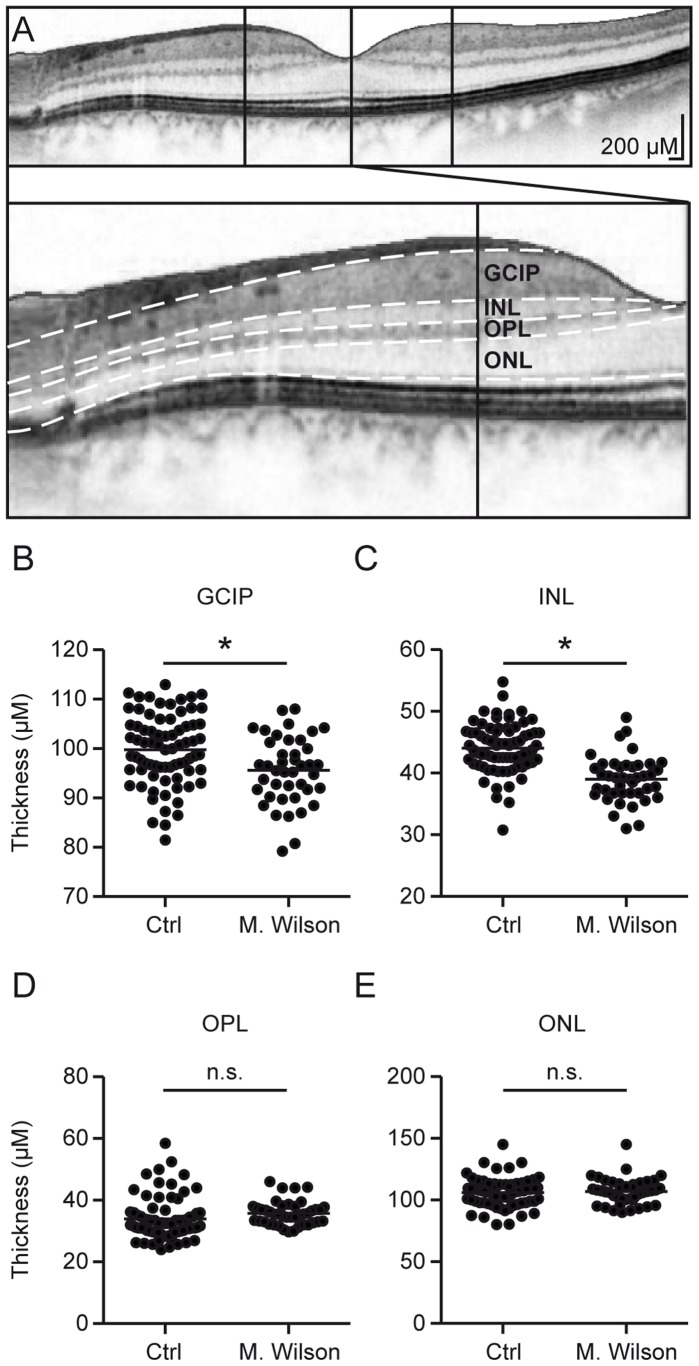
Manual segmentation: the thickness of GCIP and INL is reduced in Wilson’s disease. A The different retinal layers were manually segmented in single horizontal foveal scans and the images are displayed as negatives to better differentiate the different layers. The thickness of the different layers was measured at the vertical lines indicating the thickest point, both nasally and temporally of the fovea, except for the ONL, which was measured centrally along the vertical line. **B–E** Scatter plots of the mean thickness of the different retinal layers. Each point represents the mean of the two eyes of one patient. The mean of all patients is indicated by a horizontal bar. Significant differences are indicated by asterisks (*p<*0.05, two-tailed t test); non-significant differences are indicated as n.s.

While the results of the RNFL- and paramacular volumetric measurements were automatically analyzed by the Heidelberg Eye explorer software, the paramacular retinal layers were manually segmented as previously described [18], [30]. In brief, the segmentation of the different retinal layers in the single horizontal foveal scans was performed manually by repositioning the measurement lines (the white dotted lines in figure 2 A) on the borders between the different layers and measuring the layers at their thickest points nasally and temporally of the macula. In most subjects, the outer nuclear layer (ONL) presented only one central thickest point rather than nasal and temporal peaks as in the other layers. We therefore used the thickness at this point for analysis (the central vertical line in figure 2 A). In the few subjects with a nasal and temporal ONL thickness peaks, we used the higher value.

### Visual Evoked Potentials (VEPs)

VEPs were recorded in 27 Wilson’s disease patients and 26 control probands using monocular stimulation with a fullfield black and white checkerboard generated on a TV monitor and reversed in contrast at a rate of 3.1/s. The square size was 12 mm, which subtended a visual angle of 41 min at the subject’s eyes. VEPs were recorded from the scalp using pin electrodes positioned at Oz (active) and Fz (reference) sites. A total of 150 responses were averaged in each recording and two trials were performed for each eye. The peak latencies of N75, P100 and N140 and peak-to-peak-amplitudes from P100 to N140 were measured.

### Statistical Evaluation

Statistical analyses were performed using Microsoft Excel and Prism 5.0 (GraphPad) and SPSS Statistics 20 (IBM). To compare Wilson’s disease patients with controls, a two-tailed t-test was used and both eyes of each subject were included in the analysis as statistically dependent duplicates. ANOVA with Tukey’s post hoc test was used to compare more than two groups in the subgroup analysis.

*P-*values below 0.05 were considered significant. Correlations between two eye parameters were evaluated using the values only from the left eyes of the patients, while laboratory parameters and clinical scores were correlated with the parameters of both eyes as statistically dependent duplicates. A Pearson correlation analysis was used for normally distributed parametric values and a Spearman correlation analysis was used for nonparametric or non-normally distributed values. SPSS partial correlation analysis was performed to calculate multivariate correlations of OCT- and VEP parameters, adjusting for age, sex, laboratory parameters and clinical disease score. Subjects with missing data were excluded from the respective analysis.

The means and standard deviations are reported in the results section.

## Results

The patients and controls did not differ significantly in age or sex. The OCT findings, laboratory parameters and clinical data are shown in table 1.

### Routine OCT Parameters, RNFL Thickness and Macular Thickness

The peripapillary RNFL thickness, paramacular thickness and the thickness of the different retinal layers were measured as illustrated in figure 1A. The patients’ retinal parameters are shown in table 1. The mean peripapillary RNFL was significantly thinner compared to age and sex matched controls (Means ± standard deviation (M±SD): Wilson’s disease 95.3±8.8 µm vs. controls 99.6±10.4 µm, figure 1 A) as was the mean total macular thickness (M±SD: Wilson’s disease 311.2±15.79 µm vs. controls 321.0±14.8 µm, figure 1 B). The reduction of the macular thickness was most pronounced in the inferior quadrant and this was the only quadrant that was significantly reduced in Wilson’s disease patients compared with controls. The RNFL of our Wilson’s disease patients was more homogenously reduced and none of the quadrants alone was significantly reduced.

**Table 1:** OCT-, clinical- and laboratory parameters.

	ControlsMeans (±SD)	WDMeans (±SD)	ControlsMedian (IQR)	WD Median (IQR)	p-value	Difference (95% C.I)
Mean RNFL µm	99.6 (±10.4)	95.3* (±8.8)	100(91;107)	95(88;99)	**0.0267**	−4.27 (−4.63; −3.92)
Mean total MT µm	321 (±14.81)	311.3* (±15.8)	323(312;330)	309(301;317)	**0.0012**	−9.7 (−11.3; −8.1)
GCIP µm	99.8 (±7.1)	95.6* (±6.8)	100(96;105)	96(91;101)	**0.0026**	−4.17 (−4.63; −3.71)
INL µm	44.0 (±4.0)	39.0* (±3.7)	44(42;47)	39(37;41)	**<0.0001**	−5.04 (−5.29; −4.81)
OPL µm	33.9 (±6.8)	35.8 (±3.9)	32(30;36)	36(33;38)	0.1069	1.86 (1.5;2.22
ONL µm	106.0 (±11.3)	107.0 (±10.6)	107(99;112)	107(100;113)	0.6507	1 (1.7;0.3)
VEP N75 ms	74.0 (±5.5)	80.3* (±8.3)	74(72;77)	78(75;85)	**0.0019**	6.37 (7.41;5.32)
VEP P100 ms	103.9 (±5.2)	108.2* (±6.8)	103(99;108)	107(104;113)	**0.0111**	4.3 (3.8;5)
VEP N140 ms	141.5 (±10.1)	142.0 (±7.9)	143(134;148)	143(136;148)	0.8482	0.5 (−0.4;1.4)
VEP Amplitude µV	8.1 (±4.3)	8.4 (±3.4)	7(5.7;10.2)	7.8(5.7;11.0)	0.75	0.347 (−0.046;0.739)
Wilson Score		4.5 (±3.5)				
Disease Duration y		15.7 (±10.6)				
Follow up time y		9.8 (±5.7)				
Serum Cu^2+^, mg/l		0.35 (±0.27)				
Cu^2+^in 24 h urin mg/d		0.30 (±0.69)				
Caeruloplasmin mg/dl		8.1 (±8.5)				
Age y	42.6 (±13.2)	40.2 (±13.6)	45(31;53)	42(28;49)	0.776	
Sex male/female	29/35	18/24				

The means (± standard deviation), the p-values and the mean difference from Wilson’s disease to controls with a 95% confidence interval (95% C.I.) are indicated for the acquired parameters. The abbreviations are as follows: RNFL = peripapillary retinal nerve fibre layer thickness in µm, MT = macular thickness in µm, GCIP = retinal ganglion cell layer and inner plexiform layer measured together in µm, INL = inner nuclear layer in µm, OPL = outer plexiform layer in µm, ONL = outer nuclear layer in µm. Means that significantly differed from the control group are in bold and marked with an asterisk (*p*<0.05, two-tailed t-test).

### OCT Manual Segmentation

Due to the high resolution of the latest generation spectral-domain OCT device used in this study, we were capable of identifying the different retinal layers in transfoveal scans. We manually segmented the retinal layers in horizontal scans through the middle of the fovea and measured the thickness of the different layers (figure 2 A) as previously described [18], [30]. The results are summarized in table 1.

The retinal ganglion cell- and inner plexiform layer complex (GCIP) and the inner nuclear layer (INL) were reduced in Wilson’s disease patients (M±SD: GCIP: 95.5±0.8 µm, INL: 38.9±3.6 µm) compared with controls (M±SD: GCIP: 99.8±0.8 µm, INL: 44.1±0.5 µm) (figure 2B–C).

We observed no significant differences in the thickness of the mean outer plexiform layer (M±SD: OPL: controls 33.9±0.8 µm, Wilson’s disease 36.2±0.7 µm) or the outer nuclear layer (M±SD: ONL: controls: 106±1.3 µm, Wilson’s disease: 106±1.4 µm) (figure 2D–E).

### Visual Evoked Potentials

**Figure 3:**
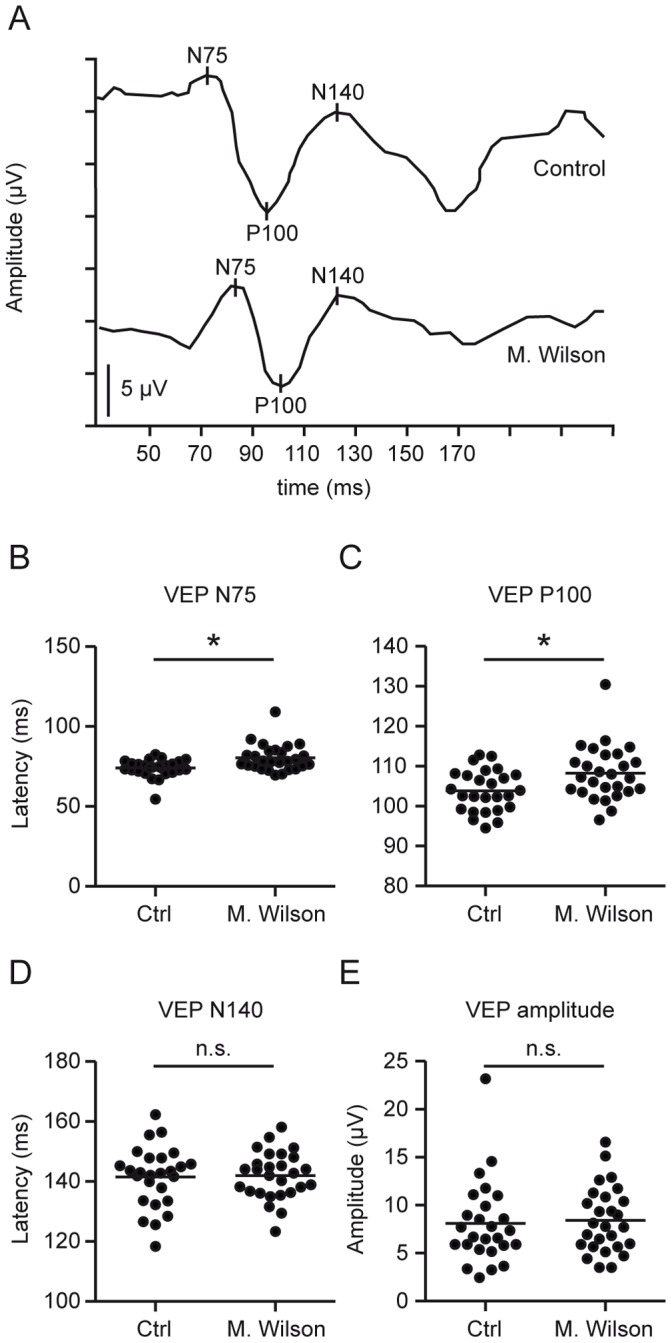
Visual evoked potentials: N75 and P100 latencies are prolonged in Wilson’s disease. A Representative VEP curves of Wilson’s disease patients and controls are displayed. **B–E** Scatter plots of the mean VEP parameters. Each point represents the mean of the two eyes of one patient. The mean of all patients is indicated by a horizontal bar. Significant differences are indicated by asterisks (*p<*0.05, two-tailed t test); non-significant differences are indicated as n.s.

The N75 and P100 latencies of the VEPs were significantly prolonged in our Wilson’s disease patients (M±SD: N75∶80.3 ms ±8.3, P100∶108 ms ±6.8) compared with controls (M±SD: N75∶74 ms ±5.5, P100∶104 ms ±5.2) while the N140 latency and the amplitude remained unchanged (M±SD: controls N140∶142 ms ±10, amplitude: 8.1 µV ±4.3; Wilson’s disease: N140 142 ms ±7.9, amplitude: 8.4 µV ±3.4). Therefore the shape of the VEP curves of Wilson’s disease patients appeared compressed (Figure 3).

### Subgroup Analysis of Treatment Groups

A subgroup analysis revealed no significant differences between patients treated with D-penicillamine, trientine, or tetrathiomolybdate for any OCT or VEP parameter (ANOVA, Tukey’s post hoc test).

### Correlations

**Figure 4:**
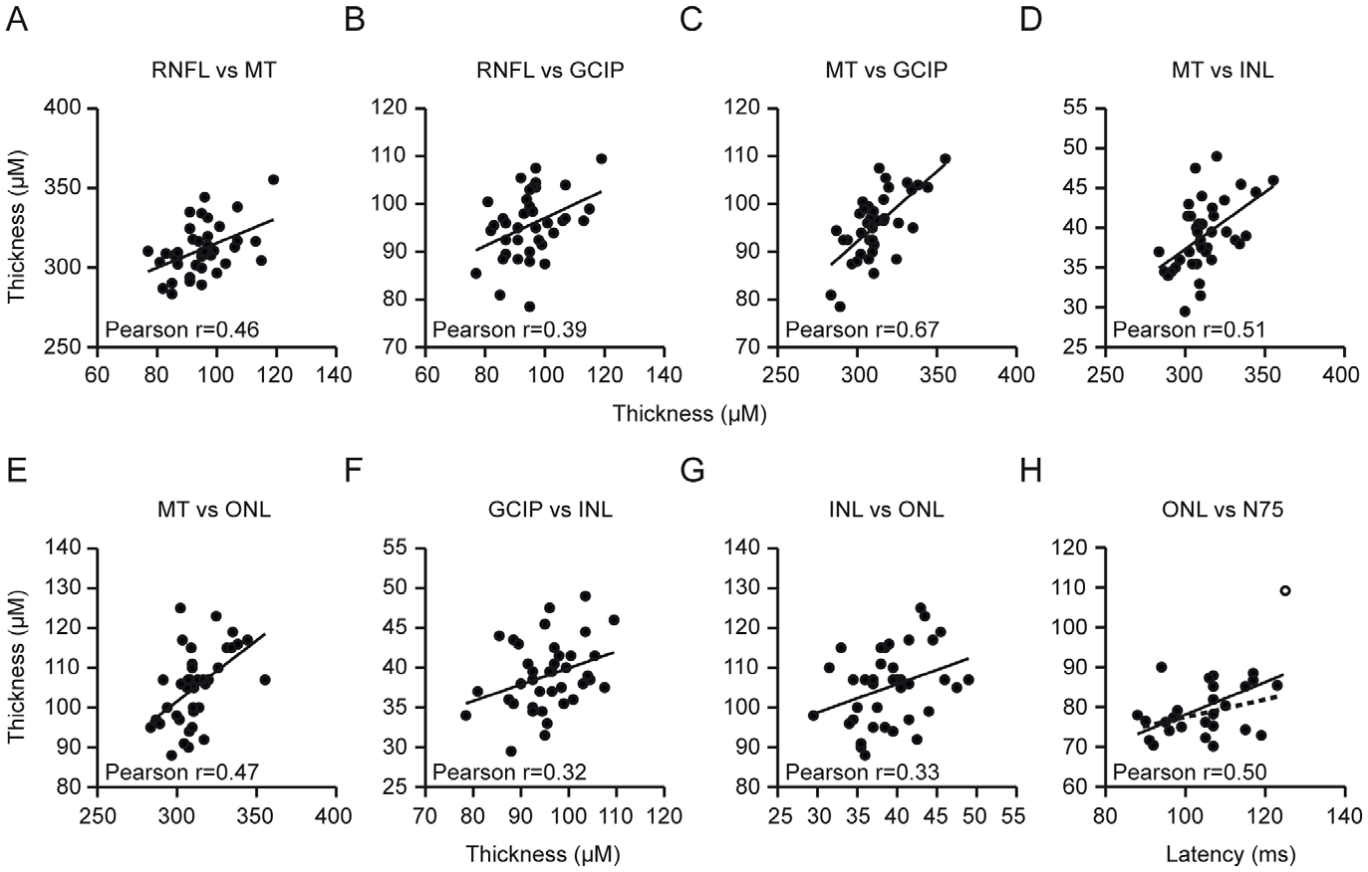
Correlations between layers. A–H The significant correlations between the thickness of the different retinal layers and the VEP parameters of the patients’ left eyes are displayed and the Pearson r is indicated (*p<*0.05, Pearson). **H** The continuous lines resemble the linear regression including, and the dotted lines excluding, an outlier with beginning hepatic failure (the outlier is marked as unfilled dot, Pearson r is indicated considering the outlier).

In our Wilson’s disease patients, the RNFL thickness correlated positively with the mean total macular thickness (*p* = 0.0031, r = 0.44, Pearson, figure 4 A) and GCIP thickness (*p* = 0.0141, r = 0.35, Pearson, figure 4 B). The mean macular thickness of Wilson’s disease patients correlated positively with the thickness of all of the macular layers except for the OPL (all *p*<0.05, GCIP: p<0.0001, r = 0.67; INL: p = 0.0008, r = 0.51; ONL: p = 0.0008; r = 0.47, Pearson, figure 4 C–E). For the manually segmented paramacular layers, we observed weak but significant positive correlations between the thickness of the GCIP and INL (*p* = 0.0398, r = 0.32, Pearson, figure 4 F) and between the INL and ONL (*p* = 0.0389, r = 0.33, Pearson, figure 4 G).

A thinner RNFL, macular thickness and GCIP appeared to be associated with longer P100 and N75 latencies and lower VEP amplitudes. However, only ONL thickness and N75 latency were significantly correlated (*p* = 0.0073, Pearson r = 0.50, figure 4 H) and the correlation was actually positive. Of note is that the ONL was not altered in Wilson’s disease patients compared with controls. We observed no significant correlation between the clinical Wilson score or the time since diagnosis of the disease and the thickness of any retinal layer or with any VEP parameter. Additionally, the Wilson score did not correlate with the time since diagnosis (Spearman).

**Figure 5:**
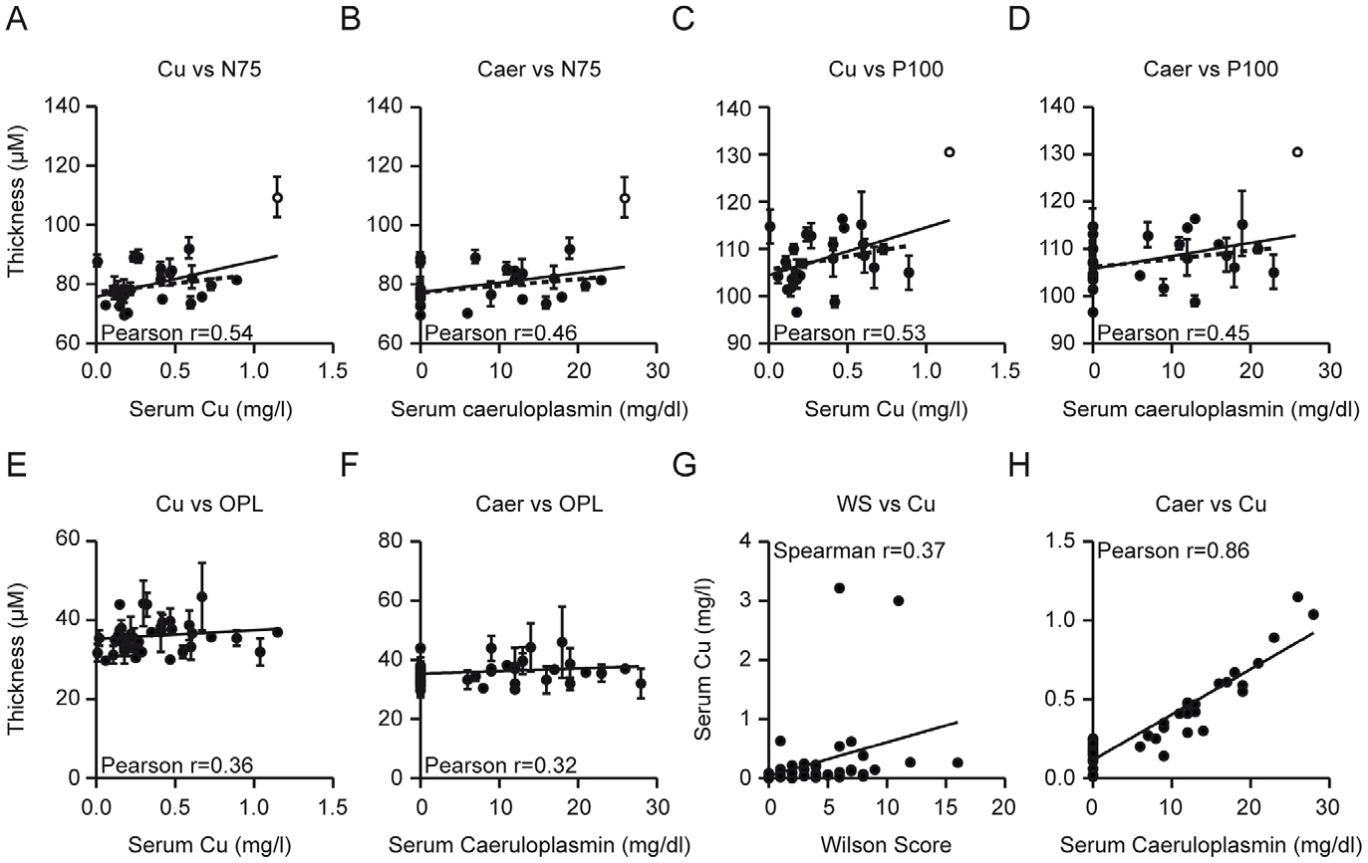
Correlations between layers, VEP parameters and laboratory. A–H The significant correlations between the laboratory parameters and the mean thickness of the retinal layers and VEP parameters of both eyes are shown and the Pearson or Spearman r is indicated (*p<*0.05 including the outlier, all comparisons were made using a Pearson analysis except for the Wilson Score, which was analyzed using a Spearman analysis). **A–D** The continuous lines resemble linear regressions including, and the dotted lines excluding, an outlier with beginning hepatic failure (the outlier is marked as unfilled dot, Pearson r is indicated considering the outlier).

An analysis of the laboratory parameters of our patients revealed weak positive correlations of both the N75 latency and the P100 latency with the concentrations of copper and caeruloplasmin in serum (N75: *p* = 0.0046 and *p* = 0.0188 respectively, both r = 0.52, Pearson, figure 5 A+B; P100: *p* = 0.0052 and, *p* = 0.0207 respectively, Pearson r = 0.52 and r = 0.45 respectively figure 5 C+D).

As all correlations of VEP parameters were very much influenced by one outlier with a N75 latency of 109 ms and a P100 latency of 130 ms, we recalculated the correlations again after removing this patient from the analysis (dotted lines in figure 4 H and 5 A–D). Without the outlier, the correlations with VEP parameters were not significant whereas P100 and N75 still differed significantly in the group comparison between Wilson’s disease and controls (t-test, *p* = 0.0019 and *p* = 0.0182, respectively).

The mean OPL thickness was weakly but significantly correlated with the concentrations of copper (*p* = 0.0181, r = 0.36, Pearson Figure 5 E) and caeruloplasmin in serum (*p*<0.05, r = 0.32, Pearson, figure 5 F). The copper concentration in 24 h urine showed a weak positive correlation with the clinical Wilson score (*p* = 0.0402, r = 0.37, Spearman, figure 5 G) and a stronger positive correlation with the caeruloplasmin concentration in serum (*p*<0.0001, r = 0.86, Pearson, Figure 5 H).

Visual acuity was above 80% in all patients and correlated significantly only with N75 and P100 VEP latency (Pearson: *p* = 0.038, r = −0.53, and *p* = 0.030, r = −0.56 respectively).

As we performed multiple correlations, we used a Bonferroni correction in a post hoc analysis to obtain a more conservative measure, reducing the risk of false positive results while increasing the risk of false negatives. After Bonferroni correction, only the correlations of mean total macular thickness with GCIP (p<0.0024), INL (p = 0.0192), and ONL (p = 0.0192) and of copper in urine with caeruloplasmin in serum (p<0.0024) remained significant.

The clinical and laboratory parameters may influence VEP- and OCT parameters. We therefore performed a multivariate correlation analysis adjusting for age, sex, the clinical disease score and the concentrations of caeruloplasmin in serum and of copper in serum and urine. When controlling for these variables, macular thickness was significantly correlated with RNFL (*p* = 0.002, r = 0.67), GCIP (*p* = 0.001, r = 0.72), INL (*p* = 0.020, r = 0.50) and ONL (*p* = 0.025, r = 0.51). RNFL was correlated with GCIP (*p* = 0.005, r = 0.611), INL (*p* = 0.028, r = 0.50) and ONL (*p* = 0.025, r = 0.511).

To test if any of the clinical parameters had influence on the retinal changes observed, we performed a linear regression based approach, analyzing the effects of age, clinical score, sex, caeruloplasmin in serum, and copper in serum and urine on macular thickness as the main retinal parameter in our patients. Of these parameters only sex had a significant (*p* = 0.026) effect with a corrected ß-coefficient of 37%. A further comparison revealed that in our collective, males indeed had a thicker macular thickness than females (*p* = 0.02 t-test; M±SD: males: 307.8 µm ±13.9, females: 318.3 µm ±16.5). In the control cohort, in contrast, we observed no difference in macular thickness between males and females (*p*>0.05, t-test, M±SD: males: 324.1 µm ±13.5, females: 318.9 µm ±15.4).

## Discussion

We were able to reproduce previously reported findings [10], [11], [14]–[16] that indicate that in Wilson’s disease, VEP latencies are delayed. We believe that the prolonged P100 latencies are likely to reflect a slowed conduction velocity of the visual tract caused by copper deposits. A structural analysis of the retina by OCT revealed reduced thickness of the RNFL, total macula and GCIP, clearly indicating pathological changes to the retinal ganglion cells and their axons in the retina. In line with previous publications [12], the VEP amplitudes of Wilson’s disease patients were unchanged compared with controls. However, in Wilson’s disease patients, low VEP amplitudes tended to be associated with thinner RNFL, GCIP and total macular thickness, although these correlations failed to reach significance. In other diseases such as multiple sclerosis, the VEP amplitude is reported to correlate with the RNFL thickness [31]. It is possible that the extent of axonal loss in Wilson’s disease patients was not sufficient to significantly reduce the VEP amplitude. However, these findings indicate that OCT may be a more sensitive parameter of axonal loss in Wilson’s disease than VEP amplitudes.

To our knowledge, no histopathological studies analyzing the retinae of patients with Wilson’s disease have been reported, so the exact mechanisms of retinal degeneration in these patients remain unclear. However, the reduction of RNFL thickness in Wilson’s disease reflects degeneration of the retinal ganglion cell axons and degeneration of the retinal ganglion cells themselves and is likely to account for the observed reduced thickness of the GCIP complex. Neuronal degeneration as a consequence of axonal damage due to copper deposition along the optic nerve and tract is a plausible explanation for these observations. The prolonged N75 and P100 latencies of VEPs indicate a slowed conduction of the visual tract due to the copper depositions themselves or secondary demyelination. The observed reduction of the total macular thickness can be attributed to the thinning of the RNFL and GCIP, which make up approximately one-third of the total thickness at the paramacular points of measurement. In line with this observation, RNFL, GCIP and total macular thickness correlated positively.

However, the reduction of the INL thickness that we observed in our Wilson’s disease patients cannot be explained as a direct consequence of copper depositions on the retrobulbar visual pathway. Either the reduction of the INL thickness is caused by a direct retinal pathology such as retinal copper deposition or it is a secondary consequence of the degeneration of the retinal ganglion cells, e.g. the bipolar cells from this layer degenerate because they can no longer signal to the degenerated retinal ganglion cells. Similar mechanisms of retrograde transsynaptic degeneration have been postulated for retinal ganglion cells in patients with hemianopia due to retrogeniculate lesions [32], [33].

It is not clear whether the treatment of our patients had any effect on the visual system or the retinal morphology. Changes of the retinal pigment epithelium under penicillamine therapy have been described in a case report from 1978 [34]. However, even though penicillamine is the most common treatment for Wilson’s disease, to our knowledge no other case of retinopathy induced by penicillamine or the other chelating agents has been reported. Furthermore, we observed no difference in any retinal parameter between patients treated with penicillamine, thrientine, or tetrathiomolybdate. We therefore do not believe that the changes of the retinal and VEP parameters are a consequence of the treatment. Of note is that most patients began their therapy with penicillamine and were later switched to another therapy due to systemic side effects, none of which involved the visual system.

Unexpectedly, we observed no significant correlations between the VEP and the OCT parameters in Wilson’s disease patients. Only the ONL thickness, which was unchanged in Wilson’s disease patients, actually correlated positively with the N75 latency. The fact that the N75 and P100 latencies correlated positively with the copper and caeruloplasmin concentrations in serum suggests that the VEP latencies may possibly be directly influenced by the current copper metabolism status at the time of examination. On the other hand, the OCT parameters that were altered in Wilson’s disease patients did not correlate with the laboratory parameters. So the thicknesses of the RNFL, GCIP, INL and total macula may be stable parameters reflecting long-term neuronal degeneration in these patients. A longitudinal analysis of these parameters is underway and will clarify this theory.

Of note is that P100 and N75 VEP -latencies were the only parameters to correlate with visual acuity as a direct measure of functional deficit.

It should be mentioned, however, that the correlations with the VEP parameters were mainly due to an outlier with a N75 latency of 109 ms and a P100 latency of 130 ms. When this outlier was removed from the analysis, none of the correlations with N75 or P100 remained significant. This was a patient who presented central serous retinopathy of his right eye, so only his left eye was included in the study. He did not report vision problems with his left eye and the ophthalmologic examination revealed no pathology of the left eye, with a corrected visual acuity of 90%. However, the laboratory parameters were indicative of a beginning hepatic failure, with changes of the liver parameters, and he was later diagnosed with a hepatocellular carcinoma. It is possible that changes of the visual pathway due to the hepatic failure, which were not accessible to the ophthalmologic exam, accounted for the prolonged VEP latencies in this patient. The case of this patient stresses the fact that marked changes in VEP latencies can be indicative of a beginning hepatic encephalopathy {Zamir, 2002 #422} and should prompt further investigations.

We observed no correlation between the OCT parameters and visual acuity using Snellen charts. To analyze the functional consequence of the structural changes observed, studies using more sensitive parameters such as analysis of the flicker fusion threshold or low contrast letter recognition are warranted.

When discussing the results of the correlations performed in our study, one must bear in mind that even though the single correlations may be significant with a *p*<0.05, the overall risk of a type I error (false positive result) increases with the number of correlations. Thus, the significant correlations should be interpreted with caution and ideally verified with an independent study. To obtain a more conservative measure of correlation we therefore performed Bonferroni corrections, though this simultaneously increased the risk of a type II error (false negative result). After the Bonferroni correction, only the correlations between macular thickness with GCIP, INL and ONL and between urine copper and serum caeruloplasmin remained significant, which is not astonishing as the macular thickness is greatly influenced by these layers and urine copper and serum caeruloplasmin concentrations are closely linked.

Using a linear regression based approach, we identified age as the only significant influence on macular thickness as the major retinal parameter, with female sex being associated with thinner macular thickness. Males and females did not differ in age, excluding an age-related artifact. Although a higher macular thickness in males compared to females has been reported before [35]–[37], the macular thickness in our control cohort did not differ between males and females. A possible explanation for the differences observed in our patients could be that the small differences between men and women, which are most likely hormone mediated, may be accentuated by the elevated copper levels in Wilson’s disease. The fact that the laboratory parameters did not serve as predictors for retinal degeneration measured by macular thickness is not at all astonishing as all patients were under therapy.

We believe that analyzing the retinal layers using OCT can provide valuable information on the ongoing neuronal degeneration in Wilson’s disease and that longitudinal evaluations are suitable for monitoring these patients. OCT and VEPs appear to be ideal tools for treatment trials and for evaluating the long-term efficacy of treatment during routine consultations. However, the manual segmentation algorithm for analysis of the deeper retinal layers used in this study is laborious and therefore not very feasible for the clinical routine. Some clinical trials have already applied fully automated segmentation techniques [17], [21], [38] that will soon be available for a wider public and may allow analysis of the deeper retinal layers in routine clinical practice.

## References

[1] Bearn AG (1953) Genetic and biochemical aspects of Wilson’s disease. Am J Med 15: 442–449. 13092113 10.1016/0002-9343(53)90134-x

[2] Cumings JN (1948) The copper and iron content of brain and liver in the normal and in hepato-lenticular degeneration. Brain 71: 410–415. 18124738 10.1093/brain/71.4.410

[3] de Bie P, Muller P, Wijmenga C, Klomp LW (2007) Molecular pathogenesis of Wilson and Menkes disease: correlation of mutations with molecular defects and disease phenotypes. J Med Genet 44: 673–688. 17717039 10.1136/jmg.2007.052746 PMC2752173

[4] Das SK, Ray K (2006) Wilson’s disease: an update. Nat Clin Pract Neurol 2: 482–493. 16932613 10.1038/ncpneuro0291

[5] Mufti AR, Burstein E, Csomos RA, Graf PC, Wilkinson JC, et al (2006) XIAP Is a copper binding protein deregulated in Wilson’s disease and other copper toxicosis disorders. Mol Cell 21: 775–785. 16543147 10.1016/j.molcel.2006.01.033

[6] Riordan SM, Williams R (2001) The Wilson’s disease gene and phenotypic diversity. J Hepatol 34: 165–171. 11211896 10.1016/s0168-8278(00)00028-3

[7] Ala A, Walker AP, Ashkan K, Dooley JS, Schilsky ML (2007) Wilson’s disease. Lancet 369: 397–408. 17276780 10.1016/S0140-6736(07)60196-2

[8] Singh P, Ahluwalia A, Saggar K, Grewal CS (2011) Wilson’s disease: MRI features. J Pediatr Neurosci 6: 27–28. 21977083 10.4103/1817-1745.84402 PMC3173909

[9] Sinha S, Taly AB, Ravishankar S, Prashanth LK, Venugopal KS, et al (2006) Wilson’s disease: cranial MRI observations and clinical correlation. Neuroradiology 48: 613–621. 16752136 10.1007/s00234-006-0101-4

[10] Arendt G, Hefter H, Stremmel W, Strohmeyer G (1994) The diagnostic value of multi-modality evoked potentials in Wilson’s disease. Electromyogr Clin Neurophysiol 34: 137–148. 8045245

[11] Das M, Misra UK, Kalita J (2007) A study of clinical, MRI and multimodality evoked potentials in neurologic Wilson disease. Eur J Neurol 14: 498–504. 17437607 10.1111/j.1468-1331.2006.01676.x

[12] Satishchandra P, Ravishankar Naik K (2000) Visual pathway abnormalities Wilson’s disease: an electrophysiological study using electroretinography and visual evoked potentials. J Neurol Sci 176: 13–20. 10865087 10.1016/s0022-510x(00)00280-x

[13] Topcu M, Topcuoglu MA, Kose G, Nurlu G, Turanli G (2002) Evoked potentials in children with Wilson’s disease. Brain Dev 24: 276–280. 12142063 10.1016/s0387-7604(02)00055-4

[14] Ilic TV, Svetl M, Petkovic S, Kostic VS (2005) [Multimodal evoked potential abnormalities in patients with Wilson’s disease]. Srp Arh Celok Lek 133: 331–337. 16623255 10.2298/sarh0508331i

[15] Hsu YS, Chang YC, Lee WT, Ni YH, Hsu HY, et al (2003) The diagnostic value of sensory evoked potentials in pediatric Wilson disease. Pediatr Neurol 29: 42–45. 13679120 10.1016/s0887-8994(03)00026-2

[16] Satishchandra P, Swamy HS (1989) Visual and brain stem auditory evoked responses in Wilson’s disease. Acta Neurol Scand 79: 108–113. 2711817 10.1111/j.1600-0404.1989.tb03720.x

[17] Saidha S, Syc SB, Ibrahim MA, Eckstein C, Warner CV, et al (2011) Primary retinal pathology in multiple sclerosis as detected by optical coherence tomography. Brain 134: 518–533. 21252110 10.1093/brain/awq346

[18] Albrecht P, Ringelstein M, Mueller A, Keser N, Dietlein T, et al.. (2012) Degeneration of retinal layers in multiple sclerosis subtypes quantified by optical coherence tomography. Mult Scler. 2012 Oct;18(10): 1422–9. Epub 2012 Mar 2. 10.1177/1352458512439237 22389411

[19] Brandt AU, Zimmermann H, Kaufhold F, Promesberger J, Schippling S, et al (2012) Patterns of Retinal Damage Facilitate Differential Diagnosis between Susac Syndrome and MS. PLoS One 7: e38741. 22701702 10.1371/journal.pone.0038741 PMC3372471

[20] Gelfand JM, Goodin DS, Boscardin WJ, Nolan R, Cuneo A, et al (2012) Retinal Axonal Loss Begins Early in the Course of Multiple Sclerosis and Is Similar between Progressive Phenotypes. PLoS One 7: e36847. 22666330 10.1371/journal.pone.0036847 PMC3359324

[21] Seigo MA, Sotirchos ES, Newsome S, Babiarz A, Eckstein C, et al.. (2012) In vivo assessment of retinal neuronal layers in multiple sclerosis with manual and automated optical coherence tomography segmentation techniques. J Neurol. 10.1007/s00415-012-6466-x 22418995

[22] Syc SB, Saidha S, Newsome SD, Ratchford JN, Levy M, et al (2012) Optical coherence tomography segmentation reveals ganglion cell layer pathology after optic neuritis. Brain 135: 521–533. 22006982 10.1093/brain/awr264 PMC3281477

[23] Walter SD, Ishikawa H, Galetta KM, Sakai RE, Feller DJ, et al (2012) Ganglion cell loss in relation to visual disability in multiple sclerosis. Ophthalmology 119: 1250–1257. 22365058 10.1016/j.ophtha.2011.11.032 PMC3631566

[24] Warner CV, Syc SB, Stankiewicz AM, Hiremath G, Farrell SK, et al (2011) The impact of utilizing different optical coherence tomography devices for clinical purposes and in multiple sclerosis trials. PLoS One 6: e22947. 21853058 10.1371/journal.pone.0022947 PMC3154907

[25] Galetta KM, Calabresi PA, Frohman EM, Balcer LJ (2011) Optical coherence tomography (OCT): imaging the visual pathway as a model for neurodegeneration. Neurotherapeutics 8: 117–132. 21274691 10.1007/s13311-010-0005-1 PMC3075740

[26] Hefter H, Arendt G, Stremmel W, Freund HJ (1993) Motor impairment in Wilson’s disease, I: Slowness of voluntary limb movements. Acta Neurol Scand 87: 133–147. 8442396 10.1111/j.1600-0404.1993.tb04092.x

[27] Roberts EA, Schilsky ML (2008) Diagnosis and treatment of Wilson disease: an update. Hepatology 47: 2089–2111. 18506894 10.1002/hep.22261

[28] Nassif N, Cense B, Park BH, Yun SH, Chen TC, et al (2004) In vivo human retinal imaging by ultrahigh-speed spectral domain optical coherence tomography. Opt Lett 29: 480–482. 15005199 10.1364/ol.29.000480

[29] Tewarie P, Balk L, Costello F, Green A, Martin R, et al (2012) The OSCAR-IB consensus criteria for retinal OCT quality assessment. PLoS One 7: e34823. 22536333 10.1371/journal.pone.0034823 PMC3334941

[30] Albrecht P, Muller AK, Sudmeyer M, Ferrea S, Ringelstein M, et al (2012) Optical coherence tomography in parkinsonian syndromes. PLoS One 7: e34891. 22514688 10.1371/journal.pone.0034891 PMC3325949

[31] Klistorner A, Arvind H, Nguyen T, Garrick R, Paine M, et al (2009) Multifocal VEP and OCT in optic neuritis: a topographical study of the structure-function relationship. Doc Ophthalmol 118: 129–137. 18779985 10.1007/s10633-008-9147-4

[32] Jindahra P, Petrie A, Plant GT (2009) Retrograde trans-synaptic retinal ganglion cell loss identified by optical coherence tomography. Brain 132: 628–634. 19224900 10.1093/brain/awp001

[33] Mehta JS, Plant GT (2005) Optical coherence tomography (OCT) findings in congenital/long-standing homonymous hemianopia. Am J Ophthalmol 140: 727–729. 16226527 10.1016/j.ajo.2005.03.059

[34] Dingle J, Havener WH (1978) Ophthalmoscopic changes in a patient with Wilson's disease during long-term penicillamine therapy. Ann Ophthalmol 10: 1227–1230. 736410

[35] Song WK, Lee SC, Lee ES, Kim CY, Kim SS (2010) Macular thickness variations with sex, age, and axial length in healthy subjects: a spectral domain-optical coherence tomography study. Invest Ophthalmol Vis Sci 51: 3913–3918. 20357206 10.1167/iovs.09-4189

[36] Wagner-Schuman M, Dubis AM, Nordgren RN, Lei Y, Odell D, et al (2011) Race- and sex-related differences in retinal thickness and foveal pit morphology. Invest Ophthalmol Vis Sci 52: 625–634. 20861480 10.1167/iovs.10-5886 PMC3053303

[37] Wexler A, Sand T, Elsas TB (2010) Macular thickness measurements in healthy Norwegian volunteers: an optical coherence tomography study. BMC Ophthalmol 10: 13. 20465801 10.1186/1471-2415-10-13 PMC2885325

[38] Yang Q, Reisman CA, Chan K, Ramachandran R, Raza A, et al (2011) Automated segmentation of outer retinal layers in macular OCT images of patients with retinitis pigmentosa. Biomed Opt Express 2: 2493–2503. 21991543 10.1364/BOE.2.002493 PMC3184859

